# Could limb laterality influence the efficacy of remote postischemic conditioning after stroke?

**DOI:** 10.1002/nep3.70048

**Published:** 2026-06-29

**Authors:** Hansen Chen, Gary K. Steinberg

**Affiliations:** ^1^ Department of Neurosurgery and Stanford Stroke Center Stanford University School of Medicine Stanford California USA

Remote ischemic conditioning (RIC) has shown promise as a neuroprotective and recovery‐oriented intervention after stroke, but clinical trial results have been inconsistent.^1^, ^2^, ^3^, ^4^ Some studies have reported improved functional outcomes, whereas others have yielded neutral results. Differences in timing, dose, treatment duration, patient population, reperfusion treatment, and outcome selection have been widely discussed. Here, we highlight an overlooked protocol variable that may have biological relevance: the laterality of limb application relative to the infarct hemisphere or paretic side.

## 1 LATERALITY PATTERNS IN EXISTING STROKE RIC TRIALS

Existing stroke RIC trials, particularly those in the acute and early subacute windows, show a potentially informative pattern in cuff laterality. Several randomized studies used unilateral RIC, although limb selection was often operational rather than mechanistically defined. RESCUE BRAIN applied lower‐limb RIC to the unaffected leg,^5^ while RECAST‐2,^6^ REPOST,^7^ RESIST,^8^ SERIC‐IVT,^9^ and the more recent SERIC‐EVT trial^10^ targeted the unaffected or non‐paretic upper limb; Hougaard et al.^11^ used a single upper limb without prespecified laterality relative to the infarct or paretic side. Where laterality was specified, the conditioned limb was consistently the unaffected, non‐paretic, or healthy side; no trial deliberately tested the paretic, ipsilesional limb as a distinct laterality strategy. Outcomes across these unilateral protocols have been mixed, ranging from feasibility‐oriented or neutral primary endpoints to favorable signals, including the improved 90‐day functional outcome reported in the SERIC‐EVT trial. Among studies using bilateral upper‐limb protocols, favorable signals have also been reported, including the small single‐center post‐thrombolysis trial by An et al. (*n* = 68)^12^ and the larger multicenter RICAMIS trial (*n* = 1893),^13^ in which bilateral upper‐limb RIC improved 90‐day excellent functional outcome. This pattern does not establish laterality as a causal determinant of efficacy, because these trials differ in timing, dose, duration, population, reperfusion treatment, control design, number of conditioned limbs, and other protocol features. Rather, it suggests that stimulation laterality may be an underreported design variable worth examining in completed and future RIC trials. To our knowledge, no published stroke RIC trial has prospectively compared ipsilesional versus contralesional limb RIC, leaving the contribution of laterality empirically undetermined.

## 2 BIOLOGICAL PLAUSIBILITY OF RIC LATERALITY

RIC laterality is biologically plausible because unilateral limb stimulation is unlikely to be neurologically neutral. Unilateral hand movement engages contralateral sensorimotor regions and can be accompanied by ipsilateral deactivation, consistent with interhemispheric interactions.^14^ More directly relevant to cuff‐based ischemia, sustained unilateral limb cuff occlusion, although not a standard RIC protocol, can rapidly modulate the excitability of the motor cortex contralateral to the conditioned limb.^15^ In chronic stroke patients, modulation of unilateral somatosensory input—for example, cutaneous anesthesia of the non‐paretic hand—can reduce interhemispheric inhibition from the contralesional to the ipsilesional motor cortex. This reduction has been associated with improved paretic‐hand performance, suggesting that lateralized peripheral input can influence post‐stroke interhemispheric balance.^16^ Moreover, in a rat focal ischemia model, capsaicin‐mediated blockade of afferent nerve signaling abolished limb remote ischemic postconditioning‐mediated neuroprotection, supporting a role for afferent nerve signaling in ischemic postconditioning, although this systemic approach does not establish limb‐side‐specific signaling.^17^ Direct neuroimaging of cortical activation during clinical RIC remains limited; nevertheless, the findings above collectively suggest that unilateral RIC may deliver a side‐dependent neural signal. Thus, bilateral RIC may differ from unilateral RIC not only in cumulative ischemic dose, but also in the spatial pattern of afferent input, a dimension that has received little attention in trial design and warrants dedicated testing.

## 3 INTERHEMISPHERIC BALANCE AS A FRAMEWORK

**Figure 1:**
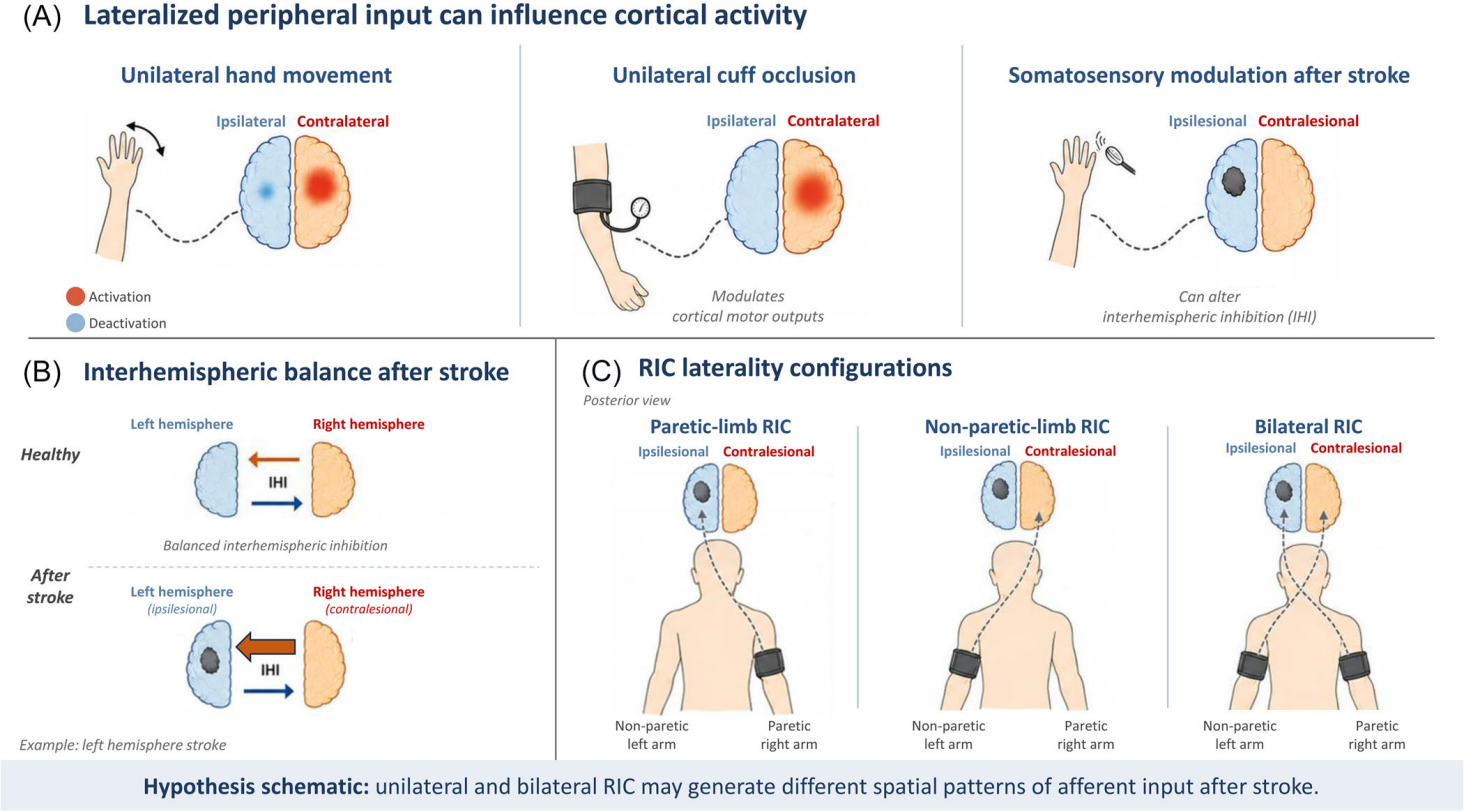
Hypothesis schematic: laterality of peripheral input, interhemispheric balance, and RIC configurations. (A) Prior studies support the biological plausibility that unilateral peripheral input can shift cortical activity, including evidence from unilateral hand movement, unilateral cuff occlusion/ischemic nerve block, and non‐paretic hand anesthesia in stroke patients. (B) After stroke, contralesional‐to‐ipsilesional interhemispheric inhibition (IHI) may become disproportionately strong, although contralesional networks can also provide compensatory support, consistent with bimodal models of motor recovery. This balance may vary across patients and post‐stroke phases. Example: left‐hemisphere stroke. (C) Because somatosensory input is represented predominantly contralaterally, paretic‐limb RIC may preferentially engage the ipsilesional hemisphere, non‐paretic‐limb RIC the contralesional hemisphere, and bilateral RIC both. Whether these spatial patterns differentially modulate post‐stroke interhemispheric dynamics, and whether the effects vary by stroke phase, remains empirically untested. This figure is an illustrative diagram, not experimental data. Some clip‐art elements were generated or refined using ChatGPT (OpenAI) and were reviewed by the authors. IHI, interhemispheric inhibition; RIC, remote ischemic conditioning.

Interhemispheric interactions provide a plausible framework for why this spatial pattern may matter after stroke. In healthy individuals, interhemispheric inhibition mediated through transcallosal pathways helps maintain balanced motor control. After a stroke, particularly when motor networks are involved, this balance may be disrupted. The contralesional hemisphere can exert excessive inhibitory influence over the ipsilesional hemisphere, a pattern associated with poorer motor performance in some patients.^18^ However, this relationship is not uniform. In patients with more extensive ipsilesional injury, contralesional networks may also provide compensatory support, consistent with bimodal models of post‐stroke motor recovery.^19^, ^20^ Within this framework, unilateral and bilateral RIC could represent biologically distinct inputs to an injured motor network (Figure 1). Whether and how their effects differ in stroke patients remains to be tested. Conceptually, paretic‐limb RIC may preferentially engage afferent input to the injured hemisphere, whereas non‐paretic‐limb RIC may preferentially engage the intact hemisphere, with effects that could be beneficial or detrimental depending on the balance between contralesional compensation and excessive transcallosal inhibition.

Moreover, interhemispheric dynamics evolve across post‐stroke phases. Cortical excitability, plasticity windows, and the balance between contralesional inhibition and compensation change over time. The optimal cuff laterality may therefore vary by stroke phase, with strategies suited to acute neuroprotection potentially differing from those suited to subacute or chronic recovery. Whether such phase‐by‐laterality interactions exist remains untested in published RIC trials. As an illustrative example, constraint‐induced movement therapy (CIMT), which restricts the healthier limb to promote activity in the impaired side, improves motor function when administered during the subacute and chronic phases of stroke recovery.^21^ However, in the acute phase, particularly at high intensity, CIMT may result in less motor improvement or potentially hinder motor recovery.^22^, ^23^ This analogy is not intended to imply shared mechanisms between CIMT and RIC, but rather to illustrate that the timing of limb‐targeted interventions relative to stroke phase can shape their effects on recovery. Testing whether RIC laterality similarly interacts with stroke phase may help inform more tailored and phase‐specific protocols.

## 4 POTENTIAL NEXT STEPS

First, existing RIC trials should be systematically reviewed at the protocol level to better characterize and quantify cuff placement and limb laterality. Although many unilateral stroke RIC protocols appear to have favored the unaffected or non‐paretic limb, the extent to which laterality was prespecified, recorded relative to infarct hemisphere, or analyzed as a treatment‐effect modifier remains unclear. Such a review should also document operational factors that may have shaped historical limb selection, including cuff tolerability, feasibility of placement on the paretic limb, delivered dose, and adherence. Where individual patient‐level data are feasibly available, laterality could then be tested as an effect modifier of RIC outcomes, while accounting for timing, dose, treatment duration, baseline severity, reperfusion therapy, and adherence.

Second, preclinical studies should directly compare ipsilesional, contralesional, and bilateral RIC in stroke models across acute, subacute, and chronic phases. Such studies could assess functional outcomes alongside mechanistic readouts of interhemispheric dynamics and afferent nerve signaling.

Third, if preclinical and systematic‐review evidence support side‐specific effects, future clinical trials could prospectively assign RIC laterality rather than treating limb choice as a practical procedural detail. A factorial or stratified design comparing ipsilesional, contralesional, and bilateral RIC could clarify whether laterality modifies treatment response and whether different strategies are optimal for neuroprotection versus recovery.

## 5 CONCLUSION

RIC remains a promising but incompletely optimized intervention for stroke. Existing trials suggest that cuff placement and limb laterality have varied across studies, yet laterality has rarely been prespecified as a mechanistic variable, recorded relative to infarct hemisphere, or analyzed as a biological variable. We propose that RIC laterality is not merely a procedural detail. Because unilateral and bilateral limb conditioning may generate different spatial patterns of afferent input, they could interact differently with post‐stroke interhemispheric dynamics across phases of injury and recovery. Testing this hypothesis, while accounting for dose, timing, and treatment duration, may help refine RIC protocols and clarify whether limb laterality represents a potential contributor to the variability in RIC effects observed across stroke trials.

## AUTHOR CONTRIBUTIONS

**Hansen Chen:** Conceptualization (equal); investigation (lead); validation (equal); funding acquisition (lead); writing—original draft (lead); writing—review and editing (equal). **Gary K. Steinberg:** Conceptualization (equal); project administration (lead); resources (lead); supervision (lead); validation (equal); funding acquisition (lead); writing—review and editing (lead).

## CONFLICT OF INTEREST STATEMENT

The authors declare no conflicts of interest.

## DATA AVAILABILITY STATEMENT

No new data were created or analyzed in this study.

## ETHICS STATEMENT

This article is a hypothesis article and did not involve new studies with human participants, animals, or identifiable human data. Therefore, ethical approval and informed consent were not required.
